# Targeting Agents in Biomaterial-Mediated Bone Regeneration

**DOI:** 10.3390/ijms24032007

**Published:** 2023-01-19

**Authors:** Miguel Gisbert-Garzarán, María Natividad Gómez-Cerezo, María Vallet-Regí

**Affiliations:** 1 Departamento de Química en Ciencias Farmacéuticas, Facultad de Farmacia, Universidad Complutense de Madrid, Instituto de Investigación Sanitaria Hospital 12 de Octubre i+12, 28040 Madrid, Spain; 2 CIBER de Bioingeniería Biomateriales y Nanomedicina CIBER-BBN, 28040 Madrid, Spain

**Keywords:** bone regeneration, nanoparticles, nanomedicine, scaffolds, SELEX, phage display, peptides, aptamers, osteoporosis, biomaterials

## Abstract

Bone diseases are a global public concern that affect millions of people. Even though current treatments present high efficacy, they also show several side effects. In this sense, the development of biocompatible nanoparticles and macroscopic scaffolds has been shown to improve bone regeneration while diminishing side effects. In this review, we present a new trend in these materials, reporting several examples of materials that specifically recognize several agents of the bone microenvironment. Briefly, we provide a subtle introduction to the bone microenvironment. Then, the different targeting agents are exposed. Afterward, several examples of nanoparticles and scaffolds modified with these agents are shown. Finally, we provide some future perspectives and conclusions. Overall, this topic presents high potential to create promising translational strategies for the treatment of bone-related diseases. We expect this review to provide a comprehensive description of the incipient state-of-the-art of bone-targeting agents in bone regeneration.

## 1. Introduction

Bone diseases are an increasing public health concern that lead to hundreds of millions of fractures every year, with osteoporosis being the main contributor to this figure. This disease reduces the life quality of patients and entails exorbitant expenses in public health. Bone diseases appear when the process of bone formation is impaired. Bone remodeling involves the continuous degradation of old bone followed by the deposition of new bone. Current treatment options are mainly based on the administration of antiresorptive agents, which inhibit the activity of the cells involved in the destruction of old bone, and only a few anabolic treatments are available. However, all those options are known to produce different side effects, many of them derived from the fact that the compounds are not selectively targeted to the bones [1].

In the last few decades, the field of biomaterials has experienced a spectacular growth, and many different types of macromaterials and nanomaterials have been applied to the treatment of uncountable diseases, including several bone diseases such as osteoporosis, osteosarcoma, Paget’s disease, and osteonecrosis, among others [2,3,4]. How these materials contribute to bone regeneration can be considered from different points of view. For instance, they can be synthesized so that their composition promotes the formation of new bone, or they can host therapeutic molecules that promote such bone regeneration. Such biomaterials can be classified as osteoinductive (promote osteogenesis, e.g., systemic treatments with nanomaterials) or osteoconductive (facilitate bone growth through a surface, e.g., implants and scaffolds) [5,6].

As-synthesized, those materials are unable to target bone tissue or bone cells, which diminishes the potential efficacy of those treatments. In this regard, there are differential features of those bone scenarios that can be employed to design structures that guide those materials toward their final fate. Such a targeting approach has been widely applied in the design of anticancer nanomedicines [7], but it has been scarcely explored in biomaterial-based bone regeneration.

**Figure 1:**
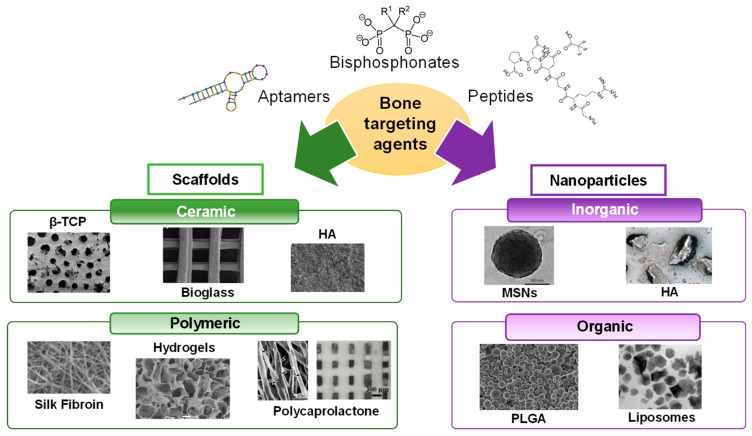
Summary of the different biomaterials that have been employed in the design of targeted systems for bone regeneration. Abbreviations: β-tricalcium phosphate (β -TCP), hydroxyapatite (HA), mesoporous silica nanoparticles (MSNs), poly (lactic-co-glycolic acid) (PLGA).

The aim of this review is to provide an overview of the different targeting agents aimed at recognizing the bone microenvironment, from small molecules to complex macromolecules. To facilitate the readability, the review was continuously organized based on whether the targeting agent employed recognizes the bone surface or specific bone cells. In addition to describing their origin, we report on the available research based on macromaterials and nanomaterials engineered with such structures for enhanced bone regeneration. In addition to those materials aimed at regenerating bone tissue, a few examples regarding cartilage regeneration employing targeted scaffolds will also be shown due to their high interest for tissue engineering and potential in this field. A summary of the biomaterials covered in this review is shown in Figure 1.

## 2. Main Features of the Bone Microenvironment

The bone microenvironment is a highly complex scenario that involves many different types of cells, components, as well as bone formation and bone resorption processes. Here, we provide a brief introduction to the main actors that make possible the process of bone remodeling and bone healing and regeneration.

### 2.1. Bone Structure

Healthy bone in adults is a composite formed by organic matrix (20–40%), inorganic mineral (50–70%), water (5–10%), and lipids (1–5%) [8]. These parameters are directly related to age, lifestyle, nutrition, and diseases [9]. Bone tissue presents three functions, namely protection of vital organs, mechanical support for locomotion, and regulation of mineral homeostasis. The correct development of these functions is directly related to the constant bone remodeling. Therefore, keeping the health of bone tissue is essential for the global metabolism.

#### 2.1.1. Organic Matrix

The bone organic matrix is mainly composed of collagen (about 90%). Type 1 collagen consists of a triple-helical molecule with two identical alpha-1 chains and a single alpha-2 chain, which are structurally similar, but genetically different [10]. It is the unique structure of collagen that allows its deposition in layers, thereby shaping mature bone.

**Figure 2:**
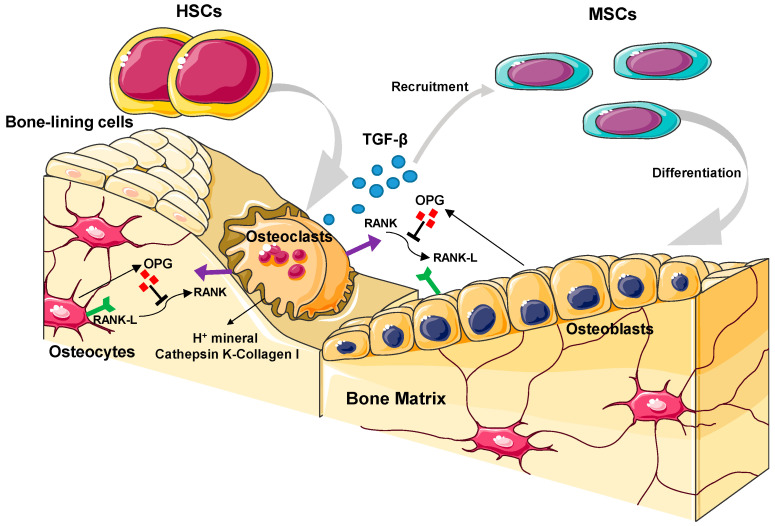
Representation of the different cell types involved in bone remodeling. The figure shows the most-relevant metabolic pathways involved in bone regeneration, both in bone-forming and bone-resorption surfaces. Abbreviations: mesenchymal stem cells (MSCs), hematopoietic stem cells (HSCs), osteoprotegerin (OPG), receptor activator of nuclear factor κ B (RANK), receptor activator of nuclear factor kappa B ligand (RANKL).

The remainder of the organic matrix is essential to maintain bone biological function. This includes non-collagenous proteins (e.g., osteonectin or osteopontin), extracellular matrix (ECM) proteins, as well as cytokines and growth factors, among which bone morphogenetic proteins (BMPs) and transforming growth factor-β (TGF-β) play a major role (Figure 2). Even though their complete physiological activity has not been fully elucidated yet, they play an essential role in the correct bone metabolism, including the regulation of osteoclast and osteoblast functions, differentiation, and cellular attachment [11].

#### 2.1.2. Inorganic Matrix

The bone mineral matrix represents more than 50% of the bone tissue volume. The inorganic matrix is mainly composed of calcium (Ca) and phosphate (P) ions nucleated to form hydroxyapatite (HA) [Ca_10_(PO_4_)_6_(OH)_2_]. The nano-crystallinity of HA is essential to maintain bone structure due to its unique mechanical properties, the crystals being ca. 20–80 nm in length and 2–5 nm in thickness. HA’s nano-crystallinity provides strength and rigidity to the skeleton, thus providing outstanding mechanical properties. In addition to the highly predominant Ca and P ions, several others are present in different amounts, including sodium, bicarbonate, citrate, potassium, magnesium, zinc, fluorite, strontium, and barium, among others [12]. Among them, carbonate impurities increase the solubility of apatite, which accelerates the release of ions for homeostasis [13,14,15,16,17]. Whenever Ca is highly demanded for any metabolic process of the organism to take place, it is obtained from the bone. Hence, efficient bone remodeling is needed to efficiently supply Ca during metabolic demand, which is a consequence of the correct communication among the different cells involved [17].

### 2.2. Bone Cell Biology

Bone is a complex living structure that is constantly adapting by its architecture and composition and shows excellent self-repairing ability [18,19]. The process of bone remodeling is essential for the maintenance of the skeleton, replacing mineral stores according to the metabolic demand, and restoring the structure under mechanical stimuli. It is estimated that 10% of the adult skeleton is renewed every year, so the cells involved in bone metabolism are constantly active. Figure 2 shows a representation of the cells involved in the intrinsically related processes of bone formation and resorption during bone remodeling and regeneration, as well as the main biochemical pathways involved.

Maintaining the balance between bone formation and bone resorption is essential in tissue remodeling, which is determinant for a healthy bone metabolism. Imbalances between both processes can lead to bone deterioration, causing pathologies such as osteoporosis [20,21]. The osteogenic process is determined by the release into the environment of various cell markers by all the cells involved. Knowledge of the mechanisms of action of osteoclasts and osteoblasts has helped to predict the material’s behavior in vivo [22,23]. Four distinctly different cell types participate in the formation, resorption, and maintenance of the bone: osteoblasts, bone-lining cells, osteoclasts, and osteocytes (Figure 2).

#### 2.2.1. Osteoblasts

Osteoblasts are cells involved in the formation of organic bone matrix, producing type I collagen, among other factors. Their functions include promoting bone formation during bone development, remodeling, and regeneration. The complex cytoskeleton of osteoblasts allows their adhesion and mobility on the bone surface, which favors interactions and coordination between them to enhance bone formation [24]. Osteoblasts are precursors of osteocytes and bone-lining cells and regulate their activities to control bone function [11,25]. They are derived from mesenchymal stem cells (MSCs) after several differentiation steps that potentially promote bone formation [26]. Several studies have shown that MSCs might play an essential role in fracture reparation by differentiating to bone-forming osteoblasts. This is the reason why many materials aim at promoting osteoblast and MSCs differentiation [27,28,29,30].

#### 2.2.2. Osteoclasts

Osteoclasts are giant multinucleated cells, differentiated from hematopoietic stem cells of the monocyte/macrophage lineage, whose function is bone resorption. Their mechanism of action is initiated by the activation signal that they receive at their cell membrane through RANKL [11]. This agent induces the fusion of pre-osteoclasts, which become multinucleated cells, and the formation of their F-actin-rich cell membrane in a wavy form. Such a membrane is responsible for the binding of osteoclasts to the bone surface, a process that is essential for bone resorption to take place [31]. After osteoclast attachment, they release a range of proteolytic enzymes (e.g., cathepsin K) and protons, which acidify the surrounded media and degrade collagen and HA in the osteoclast resorption area (Figure 2) [19]. As shown in Figure 2, OPG mediates the osteoclast–osteoblast communication, which is essential for the correct bone metabolism [19].

#### 2.2.3. Bone-Lining Cells

Bone-lining cells are inactive cuboidal-shaped quiescent bone cells present in either formative or resorptive areas of the bone surface. Bone-lining cells are morphologically flat, which allow them to extend along the inactive bone areas. They act as a barrier between osteoclasts and the bone matrix, favoring their differentiation and triggering the resorption process (Figure 2). In addition, they play a major role in keeping the anatomical structure of the bone tissue by communicating with the osteocytes and canalicular system [32]. They present very few cytoplasmic organelles and a lack of markers, which have made them difficult to study in depth, and some of their functions remain unknown [33].

#### 2.2.4. Osteocytes

Osteocytes, which are the most-abundant living cells in bone tissue, are mature osteoblasts that are surrounded by the bone matrix. Their formation mechanism from osteoblasts remains unknown. In terms of structure, they are smaller than osteoblasts, and their dendritic structure is determinant in maintaining their functions [25]. They can regulate osteoclast–osteoblast activity and maintain mineral homeostasis. In addition, they act as a mechanical sensor and are able to adapt bone tissue to external mechanical stress [34,35]. Moreover, osteocytes participate in the cellular activities of bone tissue by facilitating the exchange of nutrients and waste products through the blood vessels located inside the osteons [25,36].

## 3. Macromolecules Targeting the Bone Regeneration Microenvironment

As has been mentioned above, the bone microenvironment is a complex scenario in which different types of cells exert their functions to maintain the bone homeostasis. Ideally, the treatment should be restricted only to the target cells. Otherwise, the activity of the remaining cells might be affected, leading to side effects. In this regard, the main advantage of material-based bone regeneration treatments over administering free drugs is that those materials can be endowed with selectivity toward the bone microenvironment.

These recognition agents can be directed towards either the bone surface or specific bone cells. The former can be generally accomplished with relatively simple molecules and macromolecules, whereas the latter requires advanced combinatorial techniques.

### 3.1. Targeting the Bone Surface through Small Molecules and Macromolecules

**Figure 3:**
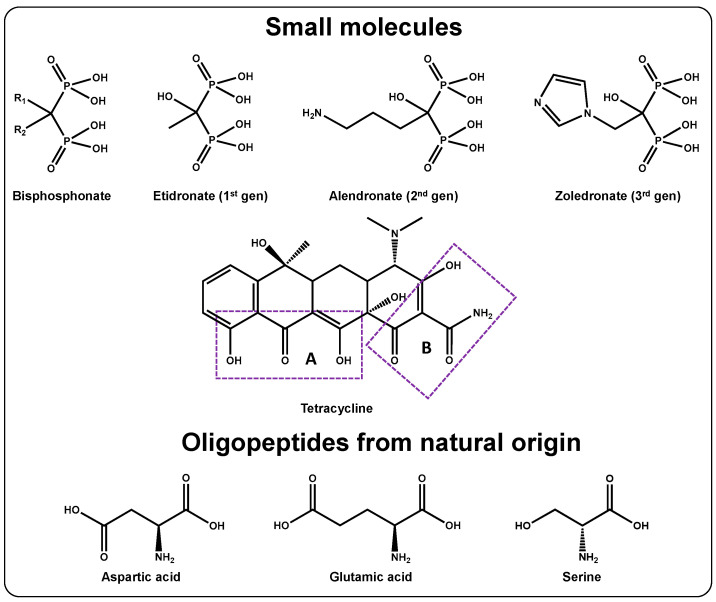
Small molecules and macromolecules that target the bone surface. Top and middle: small FDA-approved molecules. Bottom: amino acids employed in the design of oligopeptides from natural origin.

The first approach to address bone diseases is to deliver the treatment to the bone surface. This section includes therapeutic molecules and rather simple oligopeptides from protein origin that migrate to bone (Figure 3).

#### 3.1.1. Bisphosphonates

These compounds can be already found in the clinic owing to their antiresorptive features. The extent of bone targeting and biological activity depends on the residues (R1 and R2) linked to the C atom [37]. Bisphosphonates can be classified as nitrogen-free (first-generation) and nitrogen-containing (second- and third-generation) compounds (Figure 3, top), with increasing anti-resorptive activities as the R2 residue evolves toward the introduction of amine-containing groups [38].

For a given bisphosphonate, the binding process involves the formation of a tridentate complex among R1 (OH), the deprotonated oxygens of the phosphate groups, and the Ca^2+^ ions of HA [39]. The nature of R2 also influences the binding, being stronger for the nitrogen-containing generations. In this regard, each R2 residue will lead to different 3D bisphosphonate conformations. This will affect how the compound interacts with the HA surface and will determine its surface charge. It is suggested that the more positive the surface is, the more bisphosphonates will further attach to the bone, increasing the targeting ability [40]. Even though several bisphosphonates are currently in the clinic, it will be shown below that their use as targeting agents seems to be restricted to alendronate, likely because its amino group facilitates conjugation.

#### 3.1.2. Tetracyclines

Tetracyclines are a broad-spectrum family of antibiotics that are widely used in the clinic [41]. They affect both osteoblasts and osteoclasts. Regarding osteoblastic cells, high doses inhibit their proliferation, whereas low doses have an osteogenic effect [42]. Osteoclast activity is also impaired by these compounds, which can be beneficial for antiresorptive treatments. In this sense, it has been shown that tetracyclines inhibit RANKL-induced osteoclastogenesis [43] and can induce their apoptosis [44].

This class of compounds, which share a core composed of four six-membered rings, can be of natural or synthetic origin, each of them showing different functional groups [41]. The bone-targeting mechanism is based on the appearance of coordination bonds with the Ca^2+^ ions of HA, in particular by interacting with Areas A and B highlighted in Figure 3, middle [45].

#### 3.1.3. Oligopeptides from Natural Origin

The rationale behind the design of these structures is the finding that certain proteins with bone affinity present repeating units of glutamic acid (Glu) or aspartic acid (Asp) within their structure. The exact bone targeting mechanism remains unknown, although it seems to be related to the interaction with Ca^2+^ ions. Unlike bisphosphonates and tetracyclines, these structures present a safer profile. In this regard, bisphosphonates may produce hypercalcemia, ocular dysfunction, nephrotoxicity, and osteonecrosis of the jaw. Similarly, tetracyclines can yield yellow teeth and hamper the growth of skeletal tissue in children [46].

It was initially demonstrated that the hexapeptide (Asp)_6_ had great affinity toward HA, allowing the detection in the femur of a conjugated dye up to 14 days (vs. ca. 24 h for the free dye) [47]. Further studies showed that, for both Asp and Glu, the best results were obtained for *n* ≥ 6 units, with the better results occurring for *n* = 10, regardless of the amino acid [48]. In addition to those linear peptides, some authors have also reported the possibility of using dendrimer-like Asp or Glu peptides to increase the presence of carboxylic acid groups and enhance binding to HA [49,50].

Unlike bisphosphonates and tetracyclines, these structures benefit from the degree of crystallinity of HA to impart selectivity toward a specific area of the bone: high degree of crystallization for bone resorption surfaces vs. low degree of crystallization for bone-forming surfaces [51]. In this regard, it was shown that (D-Asp)_8_ oligopeptides accumulated preferentially at bone resorption areas [51]. Conversely, studies on the dentin phosphoprotein led to the development of targeting peptides composed of (AspSerSer) motifs [52], which further led to the (AspSerSer)_6_ peptide, which has been shown to bind to bone-forming surfaces [53]. The structures of the amino acids employed for the design of these structures are shown in Figure 3, bottom.

### 3.2. Generating Novel Structures to Target the Bone Microenvironment

The rationale behind these structures is the generation of random libraries of compounds, which are iteratively screened until the best candidate is obtained. This approach allows generating novel and selective macromolecules, including peptides and aptamers. The methodology for peptide generation is known as *phage display* [54], whereas that for the selection of novel aptamers is called *systematic evolution of ligands by exponential enrichment* (SELEX) [55]. The main advantage of these techniques is that, in principle, it is possible to generate a structure that shows selectivity toward a particular target while lacking affinity toward a similar one. For instance, this would translate into an osteoblast-targeted peptide that is unable to interact with macrophages or osteoclasts, thereby restricting the treatment to the target cells.

#### 3.2.1. Discovery of Novel Structures to Target the Bone Surface

Even though SELEX can be applied to the generation of HA-binding aptamers [56], most of reported targeting agents have been generated through the phage display technology. Roi et al. reported the first HA-binding peptide produced in this manner. The 12-mer peptide (SVSVGMKPSPRP) showed affinity in the micromolar range and contained two fragments that were highly conserved in the bacterial pantothenate kinase and within the bacterial glcyil-tRNA synthetase alpha subunit, respectively [57].

Gungormus et al. generated a 7-mer peptide (MLPHHGA), constrained within 2 cysteines, that not only displayed HA affinity, but also a mineralization effect. Through circular dichroism studies, the authors concluded that the peptide showed a polyproline type II secondary structure, as well as random coil or unstructured conformations. They hypothesized that the His residues could destabilize the former and lead to conformational instability, which is a feature a several bone-forming proteins [58].

Segvich et al. explored peptides for adhesion to HA and bone-like mineral, producing three 12-mer candidates with great affinity. Of them, that with the sequence VTKHLNQISQSY (known as VTK) was particularly interesting because it was similar in composition to some bone-environment-related proteins [59]. Indeed, the authors further demonstrated that phosphorylating the Ser amino acids within the structure could vastly improve the binding capacity [60].

Li et al. further looked into those proteins involved in the development of HA and sought novel HA-binding peptides based on highly conserved domains. In this manner, they employed a sidewall-displayed phage library (instead of the traditional tip-displayed phage library) to generate an 8-mer candidate (DSSTPSST) [61]. Of note, they found that the presence of proline induced a stable turn-like structure, which led to a more favorable interaction between the aspartate residues and Ca^2+^ ions.

Because proteins and peptides interact with inorganic crystals in a face-specific manner, Mao et al. generated a 7-mer peptide that could specifically bind to the {001} of HA, which is the prevalent face on the outer surface of tooth enamel. In this manner, the authors demonstrated that the sequence NNHYLPR could effectively bind to that face while inducing the precipitation of new bone [62].

Finally, a drawback of the acidic oligopeptides described in Section 3.1.3 is that they are unable to distinguish among different types of calcium-based materials. In this regard, Bang et al. screened a library of 8-mer peptides that yielded one named HA-pep3 (KNFQSRSH) as the best candidate. Not only did it show great specificity for HA over other calcium materials, but it also outperformed that of a poly(glutamic acid)*_n_*_=7_ peptide [63].

#### 3.2.2. Discovery of Novel Structures to Target Bone-Related Cells

Unlike what happened with the bone-targeted structures, both SELEX and phage display techniques have been applied to the identified novel aptamers and peptides for cell recognition. The specific purpose of these materials will be governed, at least in part, by the material to which they are grafted. Generally speaking, if they are attached to the surface of nanoparticles (NPs), they will serve as targeting agents that will help to identify the target cells once injected into the bloodstream. However, if they are anchored to the surface of scaffold-like materials, they will promote the migration and adhesion of specific cells onto their surface:

- *Aptamers*

Guo et al. carried out a SELEX study using human sarcoma osteoblasts as the target cells and HEK293 embryonic kidney cells as the negative control. They generated the aptamer O7 (5′-GAATTCAGTCGGACAGCGCACACGGAACCTCGGAACACAGCTAGCGGGGCTCACTGGATGGACGAATATCGTCTCCC-3′), which was found to interact with a specific 200 kDa protein of the osteoblast membrane [64]. Liang et al. generated the aptamer CH6 (5′-AGTCTGTTGGACCGAATCCCGTGGACGCACCCTTTGGACG-3′), which was able to target both rat and human osteoblasts, but not osteoclasts or liver cells [65]. Of note, this aptamer is so specific that it is also unable to recognize preosteoblasts or BMSCs [66].

Huang et al. discovered the aptamer J10 (5′-ACGCTCGGATGCCACTACAGGGATGGGAGGGAGGGGGCTCGTGGCGGCTAGGGGGTATAACTCATGGACGTGCTGGTGAC-3′), which showed selectivity for RAW264.7 and J774A.1 monocyte cells, but not for SVEC endothelial cells. In addition, it also recognized the THP-1 human monocyte cells [67]. Even though the authors were not aiming at achieving bone regeneration, osteoclasts can be differentiated from RAW264.7 and THP-1 cells. Hence, an antiresorptive therapy might be carried out by targeting those osteoclast precursors. However, the authors found that the J10 aptamer also recognizes circulating and cardiac cells. Hence, we would encourage using this aptamer only for bone-localized treatments.

As has been mentioned previously, stem cells have a major role in bone regeneration, the reason being that several researchers have focused on identifying specific novel aptamers. Li et al. produced an aptamer (5′-GAATTCAGTCGGACAGCGACGACGGTGATATGTCAAGGTCGTATGCACGAGTCAGAGGGATGGACGAATATCGTCTCCC-3′) that selectively identified mouse BMSCs over monocytes/macrophages and preosteoclasts [68]. Hou et al. discovered the aptamer 19S (5′-AGGTCAGATGAGGAGGGGGACTTAGGACTGGGTTTATGACCTATGCGTG-3′), which is a shorter version of the aptamer 19, which can recognize pluripotent stem cells with high specificity. They tested it against several pluripotent stem cells, using foreskin cells as a negative control [69]. Wang et al. obtained the aptamer HM69 (5′-TGCGTGTGTAGTGTGTCTGCATGCCCCTGTAATCGCCCATGGGTAGCCTCTTAGGGATTTGGGCGG-3′), which could bind mesenchymal stem cells with high selectivity, showing minor selectivity toward several other types of cells. According to the authors, this aptamer could recognize and bind the cells more effectively than the 19S one [70]. The above-mentioned aptamers were designed to target relevant cells in the skeleton. Conversely, Ardjomandi et al. focused on oral and maxillofacial applications. The authors reported the aptamer 74 (5′- GGGAGACAAGAATAAACGCTCAACAAATGGGTGGGTGTGGTGGGTGTGAAGGTGCGAGTTGATTCGACAGGAGGCTCACAACAGGC-3′), which could recognize human jaw periosteal cells. They found that it only bound the osteogenically induced cells, but not the undifferentiated or the adipogenically/chondrogenically induced ones, which would be beneficial for bone regeneration [71].

- *Peptides*

Being able to target the osteoclasts may provide a way to carry out localized, antiresorptive therapy. In this regard, Sheu et al. applied the phage display technique to generate a 12-mer peptide (TPLSYLKGLVTV) able to bind the highly expressed tartrate-resistant acid phosphatase (TRAP) within osteoclast lacunae. The authors found a peptide that showed high similarity with glypican 4, an attachment receptor found in many cells [72].

This technique can also be applied to the discovery of peptides that recognize osteoblasts. In this sense, Hoen et al. reported a 7-mer peptide (YRAPWPP) that identified KS483 osteoblasts with great affinity [73]. However, further studies against negative cells should be conducted. Sun et al. found a 5-mer peptide (SDSSD) that selectively recognized mouse and human osteoblasts, without interfering with BMSCs or osteoclasts [74]. Of note, such a sequence was also found in the dentin phosphoprotein protein, which is related to biomineralization processes.

Shao et al. identified the peptide E7, which was a 7-mer peptide (EPLQLKM) with great affinity in vitro toward BMSCs, comparable to that of the peptide RGD [75]. However, they interestingly found that, in vivo, the E7 could selectively capture such cells out of the whole set of cells within the bone marrow, which is of great interest for bone regeneration. Ramaraju et al. aimed to produce a BMSC-targeting peptide to be combined with their previously reported apatite-binding peptide [59]. In this regard, they found the 12-mer peptide DPI (DPIYALSWSGMA), which, in combination with the peptide VTK, showed improved apatite affinity and novel selectivity toward BMSCs over murine pre-osteoblasts and fibroblasts [76]. Nowakowski et al. screened directly in vivo a library of peptides on the basis that they could accumulate in the bone marrow. In this manner, they found a 7-mer peptide (STFTKSP; citing articles refer to this sequence as PFSSTKT), which the authors hypothesized might interact with the CD84 receptor of hematopoietic stem cells of the bone marrow. Based on their findings, the authors suggested that the peptide migrated specifically there, also showing accumulation in the liver, but not in an organ-specific manner [77]. In addition to those linear peptides, Sun et al. reported cyclic peptides that are selective for BMSCs. In this regard, they found the 9-mer peptides D7 (CDNVAQSVC) [78] and C7 (CTTNPFSLC) [79], which both showed greater affinity than the RGD one. However, the studies lacked a comparison with negative cells to further demonstrate the selectivity.

A summary of the seminal papers describing for the first time the above-described agents is shown in Table 1.

## 4. Targeted Nanoparticles in Bone Regeneration

The main advantage of NPs over conventional systemic treatments is that NPs allow the loading of high amounts of therapeutic payloads that, ideally, would only be released at the target area. In contrast, systemic administration of free drugs ends up with those free molecules being distributed throughout the entire body owing to their overall lack of tissue/organ/cell specificity. Regarding bone regeneration, researchers have taken advantage of some of the previously described targeting agents to engineer NPs that can selectively deliver the payload to the bone environment. Out of the large amount of bone-targeted nanocarriers available in the literature, we restricted the search to those actually demonstrating bone-regenerating features.

### 4.1. Alendronate- and Tetracycline-Modified Nanocarriers in Bone Regeneration

The simplest approach consists of modifying the NP surface with therapeutically active, bone-targeting compounds (i.e., bisphosphonates and tetracyclines). Chen et al. modified the surface of liposomal nanoparticles with alendronate to achieve high accumulation in bone. There, the NPs could transfect the stromal cell-derived factor (SDF)-1 into osteoblastic cells, producing a call effect that triggered the migration of MSCs to bone marrow to induce bone formation [80]. Guo et al. engineered magnetically responsive, alendronate-targeted PLGA polymeric NPs loaded with 17β estradiol and Fe_3_O_4_ NPs. The authors found that, upon selective accumulation in the bone, the heat generated by the applied magnetic field induced enhanced hormone release, which, in turn, ameliorated the bone loss induced in the animals [81]. Zhou et al. reported alendronate-targeted polymer vesicles able to selectively deliver β estradiol. Aside from endowing the vesicles with bone-targeting features, the authors found that alendronate acted synergistically with the released estradiol molecules, boosting bone regeneration [82].

**Figure 4:**
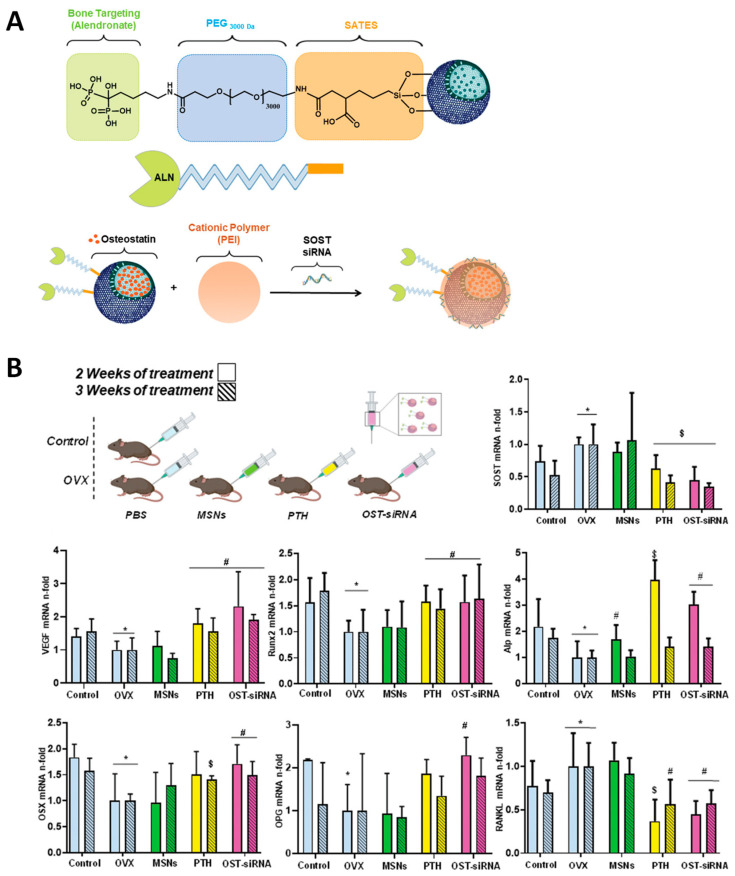
(**A**) Schematic representation of an alendronate-targeted drug delivery system based on MSNs for dual delivery of *SOST* siRNA and an osteogenic peptide. (**B**) Analysis of different osteogenic markers for each of the studied groups. Asterisks indicate *p* < 0.05 versus Control; hashtag signs indicate *p* < 0.05 versus OVX, and dollar signs indicate *p* < 0.01 versus OVX. Reproduced with permission from [83] (John Wiley and Sons).

Our group recently reported an innovative drug delivery system based on alendronate-targeted MSNs able to co-deliver the osteogenic peptide osteostatin along with *SOST* siRNA (Figure 4). Through the delivery of an anabolic + antiresorptive therapy, the NPs could synergistically improve all bone formation biomarkers. Of note, the nanosystem outperformed the FDA-approved parathyroid hormone, which is the gold standard for osteoporosis [83].

Xie et al. employed tetracyclines to engineer bone-targeted polymeric NPs for selective delivery of simvastatin, which is a lipid-lowering drug with potential antiosteoporotic features. Overall, the authors demonstrated the selective bone accumulation along with the improvement of several bone markers [84]. Tao et al. also employed tetracyclines to produce a lipid-coated nanocarrier for synergistic oral delivery of simvastatin along with calcium in a localized manner. They showed that this formulation prevented premature Ca^2+^ ions’ leakage in the gastrointestinal tract along with bone accumulation after accessing the bloodstream. Overall, the nanocarrier achieved promising osteoporotic reversion along with reduced liver injury [85].

### 4.2. Oligopeptide-Modified Nanocarriers in Bone Regeneration

**Figure 5:**
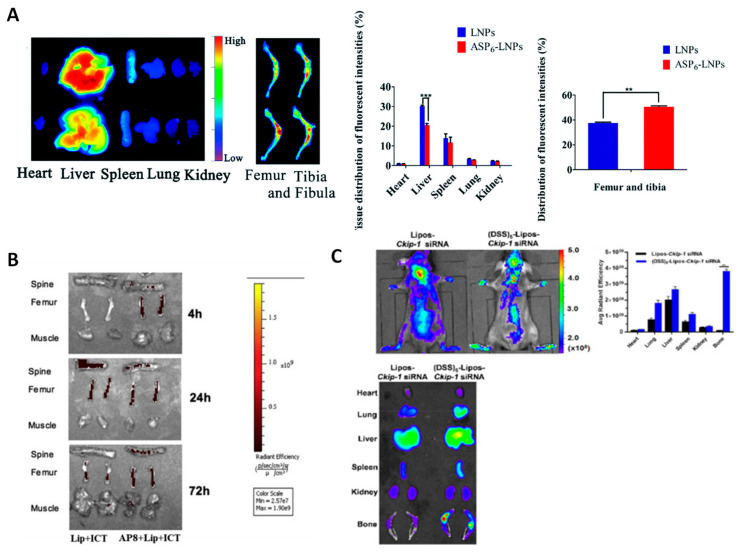
Representative images displaying the biodistribution and bone accumulation of NPs functionalized with different bone-targeting agents compared to their non-targeted counterparts. (**A**) (Asp)_6_, ** *p* < 0.01, *** *p* < 0.001. (**B**) (Asp)_8_. (**C**) (AspSerSer)_6_. Adapted with permission from [46] (The Royal Society of Chemistry), [87] (Elsevier), and [88] (Elsevier).

To the best of our knowledge, no drug delivery systems with bone regeneration capabilities based on Glu oligopeptides have been reported so far. In contrast, Asp-containing oligopeptides are more often found in the literature. In this regard, Tao et al. decorated the surface of simvastatin-loaded lipid nanoparticles with (Asp)_6_ oligopeptides, achieving enhanced accumulation in bone compared to their non-targeted counterparts (Figure 5A). The local drug release promoted osteoblastic differentiation and improved the overall bone markers [46]. Instead of decorating the surface with linear peptides, Lin et al. employed dendritic (Asp)_3_ peptides to modify the surface of PLGA NPs. The authors observed that delivering simvastatin improved the bone formation in a disuse model of osteoporosis, whereas the results obtained for the postmenopausal model were unclear [86].

As has been mentioned above, varying the number of Asp units may confer the peptide’s selectivity toward specific areas of the bone. In this sense, Huang et al. engineered (Asp)_8_-coated liposomes for icaritin delivery to bone resorption areas. Icaritin is a phytomolecule from traditional Chinese medicine with osteogenic potential. The authors found that the NPs selectively accumulated in the bone and promoted bone formation (Figure 5B). Of note, the authors found that this compound promoted the osteogenic rather than adipogenic differentiation of BMSCs [87]. Following this strategy, Sui et al. reported (Asp)_8_-targeted lipid nanoparticles to deliver an miR-21 inhibitor to osteoclasts. The authors found that silencing this miRNA inhibited osteoclastogenesis without affecting the osteoblastic parameters [89]. Similarly, Cai et al. produced (Asp)_8_-coated polyurethane nanomicelles for encapsulation of antimiRNA-214, since that nucleic acid plays a crucial role in bone remodeling. In this manner, the authors demonstrated that administering it to osteoclasts inhibited their formation on bone resorption surfaces [90].

With regard to the delivery of nanocarriers to bone-forming areas, many authors have modified their systems with the (AspSerSer)_6_ peptide. Interestingly, all research articles found for this peptide involve the application of gene therapy to reverse bone loss, either by silencing relevant structures or by supplementing the cells with relevant nucleic acids. Regarding the latter, Wang et al. demonstrated that miR-33-5p could promote the activity and mineralization of osteoblasts without affecting their proliferation. Moreover, they produced (AspSerSer)_6_-targeted liposomes bearing a miR-33-5p mimic and found that delivering them to osteopenic mice could partially reverse the induced bone loss [91]. Yang et al. modified the transfection agent *stearyl octaarginine* with the (AspSerSer)_6_ peptide to deliver a plasmid encoding the Semaphorin 3A gene. The authors found that this nanosystem could simultaneously increase the number of osteoblasts while decreasing the number of osteoclasts, obtaining overall bone gain in ovariectomized mice [92].

Several authors have proposed the delivery of siRNAs following this strategy to silence proteins that limit bone formation. In this sense, Zhang et al. engineered (AspSerSer)_6_-coated cationic liposomes to delivery Pleckstrin homology domain-containing family O member 1 (*Plekho1)* siRNA, a nucleic acid that targets casein kinase-2 interacting protein-1 (*Ckip-1*). The authors were able to knockdown the gene and demonstrated enhanced bone mass and improvement in the trabecular structure without activating the bone-resorbing cells [53]. Similarly, Gao et al. encapsulated *Ckip-1* siRNA, which could silence the production of *Ckip-1*. In this manner, the NPs accumulated in osteogenic cells, silencing the expression of *Ckip-1* and inducing the overall improvement of bone formation markers (Figure 5C) [88]. Yang et al. reported a series of gene delivery systems based on recombinant adeno-associated virus 9 targeted with (AspSerSer)_6_. In their first work, they could deliver an artificial miRNA targeting *shn3*, demonstrating enhanced bone formation thanks to increased osteoblast activity after silencing this protein [93]. Afterwards, they employed the same nanosystem to deliver an miRNA able to silence the expression of RANK and cathepsin K, which are key osteoclast regulators, achieving improved bone formation [94]. What is interesting about this piece of research is that this drug delivery system could reduce osteoclast activity while improving that of osteoblasts, even though the nanocarrier was theoretically targeted to bone-forming surfaces, which should be more enriched in osteoblasts than osteoclasts. This might alter the general understanding that (AspSerSer)_6_ peptides specifically target bone-forming areas. Further research on nanosystems carrying anti-osteoclastic therapeutics, but targeted with this peptide should be carried out in order to unravel this behavior.

### 4.3. Phage-Display-Peptide-Modified Nanocarriers in Bone Regeneration

To the best of our knowledge, only one research article has taken advantage of peptides generated through phage display to endow NPs with selectivity toward bone tissue. In this regard, Xiao et al. decorated the surface of amorphous calcium phosphate NPs with the peptide SVSVGMKPSPRP, which targeted the enamel HA surface. The authors found that the peptide could arrange the NPs into oriented arrays before transforming into crystals, which in the end produced enamel remineralization [95].

**Figure 6:**
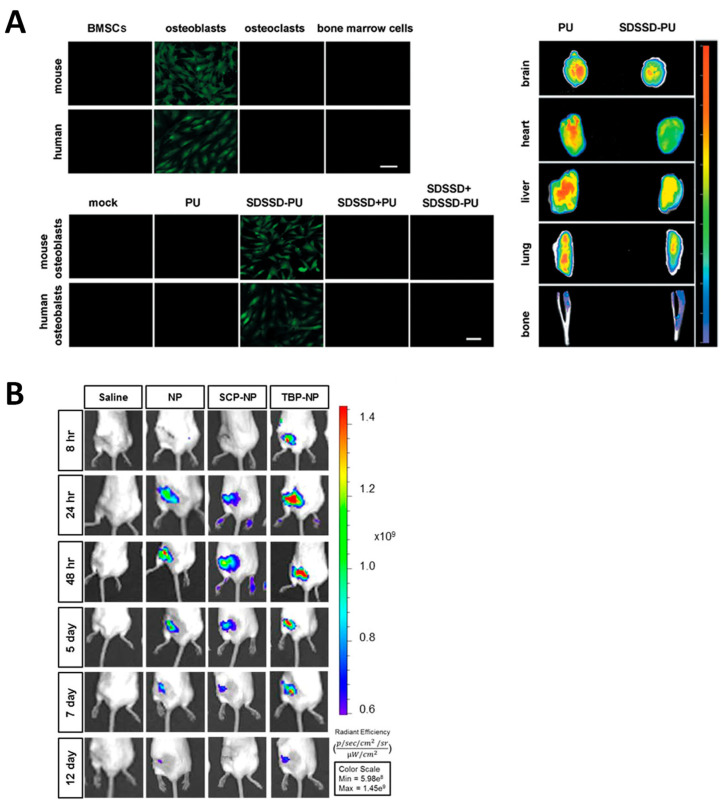
Representative images of nanocarriers targeted with peptides obtained through phage display. (**A**) Left: demonstration that SDSSD peptide targets osteoblasts over other cells. Right: biodistribution compared to the non-targeted NPs. (**B**) Biodistribution of TRAP-binding peptide-targeted NPs (TBP-NP) compared to different controls. Reproduced with permission from [74] (ACS Publications) and [97] (ACS Publications).

Regarding osteoblast-targeting peptides produced through this methodology, only nanocarriers bearing the SDSSD peptide have been reported so far. The discoverers of this fragment employed it to engineer polyurethane nanomicelles encapsulating nucleic acids. The authors found that miR-214 levels in osteoblasts decreased 80% after administering the targeted NPs and that the bone microarchitecture and bone mineral density greatly improved in the group that received the SDSSD-bearing group (Figure 6A) [74]. Taking advantage of this peptide, Cui et al. engineered an osteoblast-targeted exosome nanocarrier to deliver *Shn3* siRNA. The Schnurri-3 protein, encoded by this gene, plays a major role in bone remodeling, since it inhibits osteogenic differentiation and promotes osteoclast activity. Hence, silencing it would promote bone formation. The exosomes were secreted by MSC derived from induced pluripotent stem cells, which made themselves intrinsically antiosteoporotic, thereby producing a cooperative effect between the siRNA and the exosomes to improve bone mass [96].

Researchers have also employed the TRAP peptide generated through phage display. Wang et al. synthesized poly(styrene-*alt*-maleic anhydride)-b-poly(styrene) NPs modified with the TRAP peptide for the delivery of a β-catenin agonist able to inhibit the glycogen synthase kinase 3 beta. This protein acts as a negative regulator of the WNT pathway, consequently impairing bone formation. The authors observed that the NPs were internalized by MSCs and osteoblasts, inducing overall improvement of bone formation and bone architecture (Figure 6B) [97].

### 4.4. Aptamer-Modified Nanocarriers in Bone Regeneration

**Figure 7:**
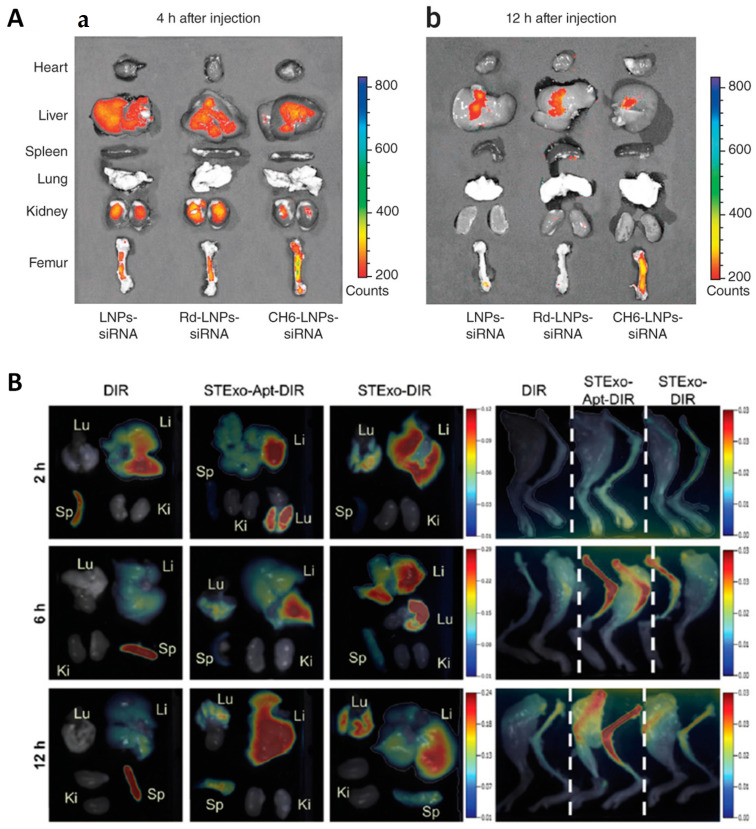
Representative images of the biodistribution of nanocarriers targeted with aptamers obtained via SELEX, compared to different non-targeted counterparts. (**A**) Osteoblast-targeted aptamer CH6. Left and right panels indicate different time points. (**B**) BMSC-targeting aptamer 5′-ACGACGGTGATATGTCAAGGTCGTATGCACGAGTCAGAGG-3. Left: non-specific accumulation in organs. Right: accumulation in the leg bones. Reproduced with permission from [65] (Springer Nature) and [99] (The Royal Society of Chemistry).

The authors that reported the osteoblast-targeting aptamer CH6 employed it to produce lipid nanoparticles for the delivery of *Plekho1* siRNA. They observed superior accumulation in bone with minimal accumulation in the liver, which boosted the formation of new bone thanks to the selective gene knockdown in osteoblasts (Figure 7A) [65]. Researchers have also taken advantage of different structures to deliver NPs to mesenchymal stem cells. The discoverers of the aptamer HM69 produced a *nanoball* made of several aptamers conjugated to each other. They observed that, upon local injection, the *nanoballs* triggered the recruitment of BMSCs, which further differentiated into osteoblasts and induced the repair of the defects introduced in the bone [70]. García-García et al. employed lipid NPs modified with the aptamer 5′-GAATTCAGTCGGACAGCGCACACGGAACCTCGGAACACAGCTAGCGGGGCTCACTGGATGGACGAATATCGTCTCCC-3′ to deliver a *Sfrp-1* silencing GapmeR. The secreted frizzled-related protein-1 (SFRP-1) protein is an antagonist of the Wnt/β-catenin pathway, which promotes bone formation. The use of the aptamer led to a four-fold increase in NP bone targeting and a ten-fold decrease in hepatic accumulation, which in the end improved the bone architecture [98]. The aptamer 5′-ACGACGGTGATATGTCAAGGTCGTATGCACGAGTCAGAGG-3′ was employed by the researchers that discovered it to functionalize the surface of bone marrow-derived exosomes, which were themselves antiosteoporotic. The authors found that conjugating the aptamer had major implications since otherwise negligible accumulation in the bone could be observed (Figure 7B) [99]. A summary of the different nanosystems that have been described in this section is shown in Table 2.

## 5. Targeted Scaffolds in Bone Regeneration

Several synthetic and natural materials have been engineered to prepare bioactive scaffolds with excellent bone regeneration features [100,101,102]. How these materials are prepared determines their physicochemical and regenerative properties. In this regard, polymers and composites that combine ceramic and polymers have been widely explored. The most frequently used polymers are polylactide (PLA) [103,104], poly(ε-caprolactone) (PCL) [105,106,107], gelatin [108,109], poly(methyl methacrylate) [110], polyurethane (PU) [111], hyaluronic acid [112], and polyvinyl alcohol (PVA) [113]. With regard to inorganic materials, calcium phosphate (CP) [114,115], β-tricalcium calcium phosphate (β-TCP) [116], hydroxyapatite (HA) [36,101,117], mesoporous bioactive glasses (MBGs) [100,118], and bioglasses [119,120,121] have been shown to present high potential to regenerate bone tissue [122,123,124]. In addition to regeneration purposes, tissue engineering is expanding its objectives to regenerate more than one tissue at the same time. Even though this review is focused on bone, several authors have increased the complexity of scaffolds to address multiple tissue regeneration. This is especially interesting to repair joint defects, as cartilage and bone form an intricate environment whose simultaneous regeneration requires a more complex approach. Indeed, some of the works described below address only chondrogenesis, and some of them focused on both bone and cartilage regeneration at the same time, opening promising strategies for joint defect reparation.

Even though several combinations have been reported in the past, it is still possible to boost the osteogenic features of these composites. In this sense, it has been shown that modifying the surface of these materials with targeting agents enables selective cell migration onto those structures, which may improve bone regeneration. In this context, only peptides and aptamers play a relevant role, since bisphosphonates have only been employed as therapeutics, rather than as targeting agents [125,126].

### 5.1. Peptide-Modified Scaffolds in Bone Regeneration

So far, the peptide RGD (arginine-glycine-aspartic acid) has been mostly employed as an inducer of bone-related cell migration onto scaffolds [103,127,128,129,130]. It has been reported that this peptide enhances osteoblast adhesion onto scaffolds, improving cell spreading and differentiation [103,130,131,132,133]. Some representative examples of RGD-decorated scaffolds are shown below.

**Figure 8:**
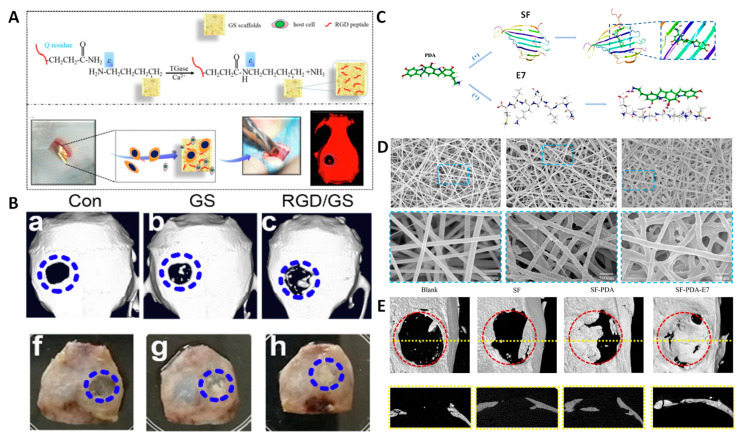
Example of systems employing the most-common peptides for bone regeneration. Left: RGD. (**A**) Fabrication scheme. (**B**) µCT images of the repaired calvarian bone after 12 weeks. Right: E7. (**C**) Preparation scheme. (**D**) Electrospun fiber morphology of different groups. (**E**) Magnetic resonance imaging (MRI) and µCT images of the repaired calvarian bone after 8 weeks. Adapted with permission from [131] (Elsevier) and [136] (ACS Publications).

Roy et al. designed scaffolds that combined in a thiolated hyaluronic acid-polyethylene diacrylate hydrogel the positive effect of RGD and the VEGF-R2 aptamer. The authors found enhanced cell migration and angiogenesis in vitro, although the study lacked in vivo validation [134]. Another system that combined bioactive molecules with RGD was proposed by Gan et al., who functionalized chitosan and β-TCP freeze-drying scaffolds with RGD and incorporated BMP in the system, finding high biocompatibility and cell adhesion and exerting a synergistic effect, as well as enhanced osteoinductive behavior in vivo [133]. The system proposed by Li et al. modified gelatin sponges through enzymatic linking, enhancing MSCs’ adhesion from skeletal muscle in an ectopic defect thanks to the RGD motif. The authors observed excellent bone regeneration after implantation of the sponges in a critical-size mouse bone (Figure 8A,B) [131].

In addition to the peptide RGD, some peptides discovered through the phage display technique have been employed in the design of bone-regenerating scaffolds. In this regard, the peptide EPLQLKM has recently attracted much attention. For instance, Zhang et al. modified Ca–alginate scaffolds with the peptides EPLQLKM and P15 (selectivity towards collagen type I). In this manner, the authors achieved improved BMMSC attachment along with the already-known capacity of P15 to enhance cell attachment, proliferation, and ECM production. Taken together, they found both osteogenic and chondrogenic differentiation, obtaining simultaneous cartilage and subchondral bone regeneration in a rabbit osteochondral defect model [135]. The osteogenic performance of this peptide sequence has also been evaluated using silk fibroin electrospun scaffolds coated with polydopamine. The presence of the peptide promoted BMSC attachment and differentiation due to its high specificity, both in vitro and in vivo (Figure 8C,D) [136]. Zhang et al. proposed PLA/gelatin scaffolds containing glycosaminoglycan, prepared with the electrospinning technique, functionalized with this peptide for osteochondral tissue engineering. The authors found that application of this system promoted both bone and cartilage regeneration, addressing the problem of joint defect regeneration. The materials bearing the peptide showed improved BMSC migration and allowed differentiation to either chondrogenic or osteogenic phenotypes in a knee osteochondral rabbit defect [137].

The osteoblast-targeting peptide SDSSD has also been employed to selectively recruit cells. Tang et al. reported SDSSD-modified cyclodextrine/chitosan 3D porous scaffolds obtained by vacuum drying [138]. The authors found that delivering free SDSSD from the scaffold induced the recruitment of osteoblasts, whereas the grafted peptide triggered macrophage migration, which changed the M1/M2 ratio to promote the M2 phenotype, reducing inflammatory response. Overall, the system was shown to enhance intramembranous ossification and improve bone regeneration in craniofacial defects.

Cyclic peptides have also been implemented into scaffolds for bone regeneration. The authors that generated the peptide C7 grafted it to β-TCP scaffolds and observed enhanced recruitment of BMMSCs in vitro, although further in vivo validation is needed [79]. The same research group validated in vivo the behavior of the peptide D7 grafted onto β-TCP scaffolds. In this case, the authors observed improved recruitment of BMMSCs, which led to clear improvement of osteonecrosis of the femoral head [139].

The peptide sequence PFSSTKT has been employed to produce materials for cartilage tissue regeneration. This application is interesting because the poor self-healing ability of articular cartilage may eventually lead to osteoarthritis if untreated. In this sense, Lu et al. prepared a composite hydrogel that combined an oriented acellular cartilage matrix with a self-assembling peptide containing the mentioned sequence. The authors found specific migration of endogenous stem cells and subsequent chondrogenic differentiation, observing full recovery of the cartilage defect after 3 and 6 months post-surgery [140]. Similarly, Huang et al. modified chondrocyte extracellular matrix particles with this peptide and combined them with GelMA hydrogel, creating an excellent environment for chondrogenic differentiation. They demonstrated that the targeted group triggered the highest recruitment of BMMSCs, observing improved efficiency in repairing the cartilage of the rabbits [141].

Wang et al. prepared β-TCP scaffolds functionalized with the peptide DPIYALSWSGMA and demonstrated in vitro that it could increase the recruitment of mouse BMMSCs, compared to the untargeted counterpart [142]. The same research group employed those targeted scaffolds to heal osteonecrosis of the femoral head. In addition to being selective toward mice BMMSCs, the authors demonstrated the peptide’s affinity toward rabbit BMMSCs. The targeted group could improve the condition of the animals, outperforming the core decompression technique that is commonly employed for this disease [143]. Ramaraju et al. engineered an innovative version of this peptide, merging in a single structure cell-targeting features with HA targeting thanks to the peptide VTKHLNQISQSY. In this manner, they employed one side to attach the peptide to the surface of a bone-like mineral and the other one to recruit the migration of induced pluripotent MSCs. The authors took advantage of this feature to coat the bone-like mineral with such cells to then implant it into the animals, outperforming the clinically available treatment in terms of bone formation [144]. Table 3 shows a summary of the materials that have been modified with peptides generated through phage display

### 5.2. Aptamer-Modified Scaffolds in Bone Regeneration

As mentioned in Section 2, several aptamers present the potential to enhance cartilage and bone regeneration through targeting or recruitment of bone cells. However, their application in combination with scaffolds remains limited, and only some of them have been validated in combination with materials.

Wang et al. prepared macro-mesoporous bioactive glass scaffolds functionalized with reduced graphene oxide and further decorated with the aptamer CH6. The authors found that this cell-free approach was able to heal the large bone defects in the femur thanks to the specific osteoblast recruitment and scaffold-mediated differentiation [145]. Yang et al. recently employed the aptamer HM69 for cartilage regeneration in a knee joint defect. It was conjugated to a decellularized cartilage extracellular matrix, which was further mixed with gelatin methacrylate. Then, they printed the bioink in combination with PCL to improve the mechanical strength. Overall, the authors observed that the composite triggered the chondrogenic differentiation of MSCs, boosting cartilage repair [146]. The same authors that reported the aptamer 74 modified the surface of β-TCP scaffolds with this aptamer. Even though the studied lacked in vivo validation, they found increased adhesion of jaw periosteal cells in vitro, which might be promising for maxillofacial regeneration [147].

In addition to those aptamers, the 19S has been the most widely used because it specifically recognizes and captures MSCs, which allows the design of systems that can not only enhance bone regeneration, but also chondrogenesis, paving the way for the design of innovative systems for articulation repair. Even though most systems involve 3D scaffolds, 2D systems can also be employed for cell recruitment and regeneration. In this regard, Wei et al. presented a Ti implant coated with hydroxyapatite that was further coated with hyaluronic acid functionalized with this aptamer. The authors found that this coating improved bone formation in the periphery of the implant [148].

**Figure 9:**
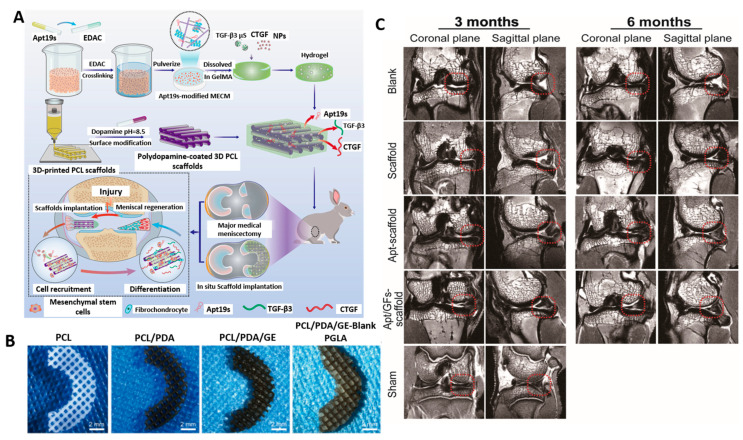
Example of photocrosslinkable bioink combined with PCL and functionalized with the aptamer 19S and growth factor. (**A**) Fabrication scheme. (**B**) Images of final scaffolds’ structure. (**C**) Magnetic resonance imaging (MRI) of the different groups. Adapted with permission from [151] (Wiley).

Regarding the use of 3D scaffolds, Hu et al. reported an innovative aptamer 19S-decorated, graphene-oxide-based scaffold aimed at inducing both osteogenic and chondrogenic differentiation. For that purpose, they engineered two layers of different composition, one targeting the cartilage and the other one targeting the subchondral bone defect. In this manner, they achieved chondrogenesis (thanks to delivering a stimulating factor) and osteogenesis of MSCs, obtaining overall improvement of the osteochondral defect [149]. Wang et al. provided an alternative system to obtain cartilage regeneration. The authors reported silk-fibroin-based scaffolds functionalized with this aptamer. The inner part of the scaffold contained hyaluronic acid to improve the chondrogenic capacity. In this manner, they found that this composition without any additional aid could improve an osteochondral defect [150]. Similarly, Li et al. prepared an aptamer-19S-containing scaffold based on GelMA/PCL for meniscus regeneration (Figure 9). The scaffolds further contained PLGA nanoparticles and microparticles encapsulating the connective tissue growth factor or transforming growth factor. Incorporating the aptamer triggered the recruitment of endogenous stem/progenitor cells, which underwent meniscogenic differentiation upon factor release from the polymeric carriers. Taken together, this multifunctional system improved neomeniscal formation in rabbits [151]. Table 4 summarizes the research articles involving scaffolds modified with aptamers for bone and osteochondral regeneration.

## 6. Conclusions and Future Perspectives

The overall goal of this review manuscript was to expose in a comprehensive manner the different structures that can be employed to target the bone microenvironment. As has been shown, some of those structures have been already implemented into NPs or macroscopic scaffolds, achieving enhanced accumulation in the bone microenvironment or selective recruitment of bone-related cells. Overall, the available literature demonstrates that such a strategy may provide a tool to improve the bone regeneration features of those materials. Nonetheless, this is a field yet to be developed, and we envision that hundreds of research articles employing this methodology will be published in the upcoming years. Because of the nature of the phage display and SELEX techniques, we anticipate that many other highly selective targeting structures will be reported in the next few years. Overall, targeting the bone microenvironment is a highly promising strategy for improved bone regeneration, which might shorten the bench-to-bedside gap in a few years.

## Figures and Tables

**Table 1:** Seminal papers reporting relevant bone-targeting agents for the first time.

Targeting Agent	Target	Ref.
*Oligopeptides from natural origin*		
D_6_	HA	[47]
D_8_	Bone resorption areas	[51]
(DSS)_6_	Bone formation areas	[53]
*Peptides generated through phage display*		
SVSVGMKPSPRP	HA	[57]
MLPHHGA	HA	[58]
VTKHLNQISQSY	HA	[59]
DSSTPSST	HA	[61]
NNHYLPR	HA ({001} face)	[62]
KNFQSRSH	HA	[63]
TPLSYLKGLVTV	TRAP	[72]
YRAPWPP	KS483 osteoblasts	[73]
SDSSD	MC3T3-E1 preosteoblastshFOB human osteoblasts	[74]
EPLQLKM	Human BMMSCs	[75]
DPIYALSWSGMA	Human bone marrow stromal cells	[76]
STFTKSP	Hematopoietic stem cells/Mouse BMSCs	[77]
CDNVAQSVC	Mouse BMMSCs	[78]
CTTNPFSLC	Rat BMMSCs	[79]
*Aptamers generated through SELEX*		
5′-CAGGGCGCTACGGTATGTGTTGGGTCTGGCGTAGGGCTGGC-3′	HA	[56]
5′-GAATTCAGTCGGACAGCGCACACGGAACCTCGGAACACAGCTAGCGGGGCTCACTGGATGGACGAATATCGTCTCCC-3′	SAOS-2	[64]
5′-AGTCTGTTGGACCGAATCCCGTGGACGCACCCTTTGGACG-3′	Rat primary osteoblasts	[65]
5′-ACGCTCGGATGCCACTACAGGGATGGGAGGGAGGGGGCTCGTGGCGGCTAGGGGGTATAACTCATGGACGTGCTGGTGAC-3′	Raw264.7J774A.1	[67]
5′-GAATTCAGTCGGACAGCGACGACGGTGATATGTCAAGGTCGTATGCACGAGTCAGAGGGATGGACGAATATCGTCTCCC-3′	Mouse BMMSCs	[68]
5′-AGGTCAGATGAGGAGGGGGACTTAGGACTGGGTTTATGACCTATGCGTG-3′	Human PSCs	[69]
5′-TGCGTGTGTAGTGTGTCTGCATGCCCCTGTAATCGCCCATGGGTAGCCTCTTAGGGATTTGGGCGG-3′	Human PSCs	[70]
5′- GGGAGACAAGAATAAACGCTCAACAAATGGGTGGGTGTGGTGGGTGTGAAGGTGCGAGTTGATTCGACAGGAGGCTCACAACAGGC-3′	Human jaw periosteal cells	[71]

Peptides: A: alanine: C: cysteine; D: aspartic acid; E: glutamic acid; F: phenylalanine; G: glycine; H: histidine; I: isoleucine; K: lysine; L: leucine; M: methionine; N: asparagine; P: proline; Q: glutamine; R: arginine; S: serine; T: threonine; V: valine; W: tryptophan; Y: tyrosine. Aptamers: A: adenine; C: cytosine; G: guanine; T: thymine.

**Table 2:** Summary of the research articles exposed in Section 3 classified according to the targeting agent employed.

Description	In Vivo Model	Ref.
*Alendronate*		
Liposomes that transfect the SDF-1 into osteoblastic cells to trigger the migration of MSCs to the bone marrow	C57BL/6 mice	[80]
PLGA NPs loaded with 17β estradiol that is released upon heat generation after application of a magnetic field	OVX SD rats	[81]
Polymer vesicles carrying β estradiol that acts synergistically with the targeting bisphosphonate	OVX SD rats	[82]
MSNs carrying an osteogenic peptide and an *SOST* siRNA that exert synergistic osteogenic effect	OVX C57BL/6 mice	[83]
*Tetracycline*		
Polymeric NPs that deliver simvastatin locally in the bone	OVX SD rats	[84]
Lipid-coated nanocarrier for the delivery of Ca^2+^ ions and simvastatin	OVX ICR mice	[85]
*(Asp)_n_*		
(Asp)_6_-coated lipid NPs loaded with simvastatin	OVX ICR mice	[46]
Dendritic (Asp)_3_-PLGA NPs loaded with simvastatin	OVC SD rats and Disuse SD rats	[86]
(Asp)_8_-coated liposomes carrying icaritin that promote osteogenic, rather than adipogenic differentiation of BMSCs	OVX C57/BL6 mice	[87]
(Asp)_8_-coated lipid NPs carrying an miR-21 inhibitor to inhibit osteoclastogenesis	OVX C57/BL6 mice (WT and miR-21-defficient)	[89]
(Asp)_8_-coated polyurethane nanomicelles for the delivery of antimiRNA-214 to inhibit osteoclastogenesis	OVX C57/BL6 mice	[90]
*(AspSerSer)_6_*		
Liposomes loaded with an miR-33-5p mimic to promote activity and mineralization of osteoblasts	Hindlimb unloading C57/BL6 mice	[91]
Transfecting agents carrying a plasmid encoding the Semaphorin 3A gene to increase the number of osteoblasts and reduce that of osteoclasts simultaneously	OVX Kunming mice	[92]
Cationic liposomes for the delivery of *Plekho1* siRNA to osteoblasts	OVX SD rats	[53]
Liposomes encapsulating *Ckip-1* siRNA to deliver it to osteoblasts	OVX C57/BL6 mice (WT and *Ckip-1* knockdown)	[88]
Adeno-associated virus 9 loaded with a miRNA targeting *shn3* in osteoblasts	OVX BALB/cJ (*Shn3^−/−^*) and C57BL/6J (*Shn3^fl/fl^*) mice	[93]
Adeno-associated virus 9 delivering an miRNA able to silence RANK and cathepsin K expression	OVX BALB/cJ and C57BL/6J mice	[94]
*Peptides generated through phage display*		
SVSVGMKPSPRP-coated amorphous calcium phosphate NPs to target enamel HA surface	-	[95]
SDSSD-coated polyurethane nanomicelles carrying antimiR-214 to silence it in osteoblasts	OVX mice	[74]
SDSSD-coated exosomes derived from pluripotent stem cells to deliver *Shn3* siRNA to osteoblasts	OVX C57BL/6J mice	[96]
TRAP peptide-coated polymeric NPs to deliver a β-catenin agonist able to inhibit the glycogen synthase kinase 3 beta	Fracture model in BALB/c mice	[97]
*Aptamers generated through SELEX*		
CH6-targeted lipid NPs for the delivery of *Plekho1* siRNA to osteoblasts	OVX SD rats	[65]
Assembly of HM69 aptamer into *nanoballs* to trigger the recruitment of BMSCs	Defect created in SD rats	[70]
Lipid NPs modified with the aptamer 5′-GAATTCAGTCGGACAGCGCACACGGAACCTCGGAACACAGCTAGCGGGGCTCACTGGATGGACGAATATCGTCTCCC-3′ to deliver a *Sfrp-1* silencing GapmeR to mouse BMSCs	OVX FVB mice	[98]
Bone marrow-derived exosomes with antiosteoporotic features functionalized with the aptamer 5′-ACGACGGTGATATGTCAAGGTCGTATGCACGAGTCAGAGG-3′	OVX C57BL/6J mice	[99]

OVX: ovariectomized; SD: Sprague-Dawley; ICR: Institute of Cancer Research; BALB: Bagg and Albino; WT: wild-type.

**Table 3:** Summary of the research articles that describe the use of phage-display-peptide-decorated scaffolds for bone regeneration.

Description	In Vivo Model	Ref.
*EPLQLKM*		
Scaffolds functionalized with this peptide and with the peptide P15 that achieve simultaneous cartilage and subchondral bone regeneration in rabbit osteochondral defect model	NZ rabbit	[135]
Silk fibroin electrospun scaffolds coated with PDA to induce osteogenic differentiation of BMSCs	SD rats	[136]
PLA/gelatin scaffolds containing glycosaminoglycan for improved BMSC migration and differentiation to either chondrogenic or osteogenic phenotypes in a knee osteochondral defect	NZ rabbit	[137]
*CTTNPFSLC*		
β-tricalcium phosphate scaffolds that enhance the adhesion, expansion, and proliferation of BMSCs	SD rats	[76]
*CDNVAQSVC*		
β-TCP scaffolds that enhance BMMSC recruitment with potential application in osteonecrosis treatment	ONFH/NZ rabbit	[139]
*DPIYALSWSGMA*		
β-TCP scaffolds that demonstrate enhanced BMMSC adhesion and proliferation	-	[142]
β-TCP scaffolds that recruit BMMSCs and improve osteonecrosis of the femoral head	NZ rabbit	[143]
Bone-like mineral functionalized with a dual-peptide containing also the mineral binding sequence VTKHLNQISQSY, which improves bone regeneration thanks to recruiting iPS cells	NIH-Lyst^bg-J^Foxn1^nu^Btk^xid^, Charles Rivers mice	[141]
*SDSSD*		
Chitosan scaffolds bearing this peptide grafted and loaded for OB recruitment and promotion of M2 macrophage polarization	SD rats	[138]
*PFSSTKT*		
Hydrogel combining an oriented acellular cartilage matrix with a self-assembling peptide containing the mentioned sequence for specific migration of endogenous stem cells and subsequent chondrogenic differentiation	NZ rabbit	[137]
GelMA hydrogel containing chondrocyte extracellular matrix particles decorated with this peptide for the recruitment of BMMSCs and chondrogenic differentiation	NZ rabbit	[138]

**Table 4:** Summary of the research articles that describe the use of aptamer-decorated scaffolds for bone regeneration.

Description	In Vivo Model	Ref.
*CH6*		
Macro-mesoporous bioactive glass scaffolds functionalized with reduced graphene oxide for specific osteoblast recruitment and scaffold-mediated differentiation for bone defect regeneration	SD rats	[145]
*HM69*		
GelMA/ PCL scaffolds for recruitment and chondrogenic differentiation of MSCs for cartilage repair	C57/BL6 mice	[146]
*74*		
β-TCP scaffolds’ potential enhances JPC cell adhesion on 3D constructs and mineralization on 2D surfaces	-	[147]
*19S*		
Bioactive titanium implants for recruitment and differentiation of BMMSCs for formation of new bone	SD rats	[148]
Graphene-oxide-based scaffold engineered in two layers of different composition, one targeting the cartilage and the other one targeting the subchondral bone defect for efficient repair of osteochondral defect	SD rats	[149]
Silk-fibroin-based scaffolds containing hyaluronic acid to improve the chondrogenic capacity for efficient repair of osteochondral defect.	NZ rabbits	[150]
GelMA/PCL scaffold containing PLGA microparticles and nanoparticles loaded with different factors for improved meniscus regeneration	SD rats/NZ rabbits	[151]
